# 2-[(6-Bromo-2-pyrid­yl)amino]pyridine *N*-oxide

**DOI:** 10.1107/S1600536808022058

**Published:** 2008-07-19

**Authors:** Yun-Hua Chen, Yin-Qiu Liu, Xi-Rui Zeng

**Affiliations:** a Zhejiang Hisun Pharmaceutical Co. Ltd, JiangXi Province Key Laboratory of Coordination Chemistry, 343009 Ji’an, JiangXi, People’s Republic of China; b College of Chemistry and Chemical Engineering, JiangXi Province Key Laboratory of Coordination Chemistry, JingGangShan University, 343009 Ji’an, JiangXi, People’s Republic of China

## Abstract

In the crystal structure of the title compound, C_10_H_8_BrN_3_O, the dihedral angle between the two pyridine rings is 2.48 (2)°. A weak intramolecular N—H⋯O hydrogen bond is present.

## Related literature

For similar structures, see: Wu (2007 ▶); Liu & Wen (2007 ▶).
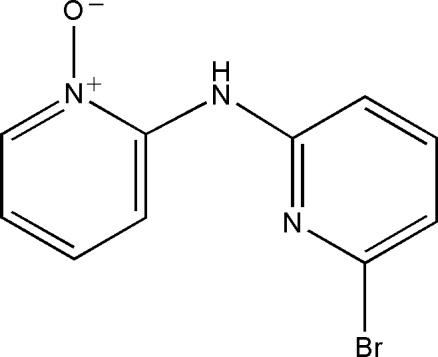


## Experimental

### 

#### Crystal data

C_10_H_8_BrN_3_O
*M* *_r_* = 266.09
Monoclinic, 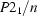

*a* = 13.402 (3) Å
*b* = 5.3016 (10) Å
*c* = 14.562 (3) Å
β = 103.498 (3)°
*V* = 1006.1 (4) Å^3^
*Z* = 4
Mo *K*α radiation
μ = 4.06 mm^−1^
*T* = 298 (2) K
0.52 × 0.13 × 0.11 mm

#### Data collection

Bruker SMART CCD area-detector diffractometer
Absorption correction: multi-scan (*SADABS*; Bruker, 1997 ▶) *T*_min_ = 0.545, *T*_max_ = 0.650
5867 measured reflections
1850 independent reflections
1263 reflections with *I* > 2σ(*I*)
*R*_int_ = 0.049

#### Refinement

*R*[*F* ^2^ > 2σ(*F* ^2^)] = 0.029
*wR*(*F* ^2^) = 0.054
*S* = 0.98
1850 reflections
139 parameters
1 restraint
H atoms treated by a mixture of independent and constrained refinement
Δρ_max_ = 0.40 e Å^−3^
Δρ_min_ = −0.28 e Å^−3^

### 

Data collection: *SMART* (Bruker, 1997 ▶); cell refinement: *SAINT* (Bruker, 1997 ▶); data reduction: *SAINT*; program(s) used to solve structure: *SHELXS97* (Sheldrick, 2008 ▶); program(s) used to refine structure: *SHELXL97* (Sheldrick, 2008 ▶); molecular graphics: *SHELXTL* (Sheldrick, 2008 ▶); software used to prepare material for publication: *SHELXTL*.

## Supplementary Material

Crystal structure: contains datablocks I, global. DOI: 10.1107/S1600536808022058/nc2103sup1.cif

Structure factors: contains datablocks I. DOI: 10.1107/S1600536808022058/nc2103Isup2.hkl

Additional supplementary materials:  crystallographic information; 3D view; checkCIF report

## Figures and Tables

**Table 1:** Hydrogen-bond geometry (Å, °)

*D*—H⋯*A*	*D*—H	H⋯*A*	*D*⋯*A*	*D*—H⋯*A*
N2—H2*A*⋯O1	0.899 (10)	2.12 (2)	2.542 (3)	107.8 (19)

**Fig. 1.**
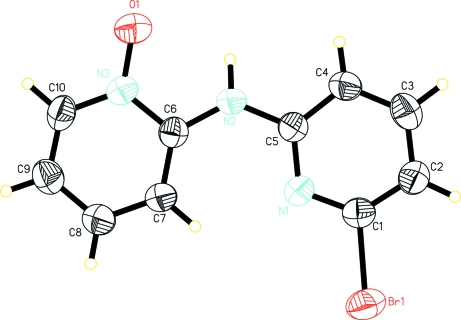
The molecular structure of (I) with labelling and displacement ellipsoids drawn at the 50% probability level.

**Fig. 2.**
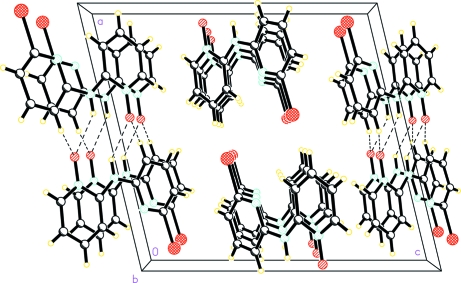
Crystal structure of the title compound, viewed along the c axis. Hydrogen bonding is shown as dashed lines.

## supplementary crystallographic information

### Comment

The structure determination was performed as a part of a project on the synthesis, structures and properties of new polypyridylamine N-oxides. In the crystal structure both pyridine rings are nearly coplanar with an dihedral angle of 2.48 (2) /%. The molecules are connected into dimers via intermolecular N-H···O hydrogen bonding and the dimers are stacked in the direction of the crystallographic b-axis in a sandwich-herringbone structure.

### Experimental

A mixture of 2.56 g 6-bromo-*N*-(pyridin-2-yl)pyridin-2-amine (0.01 mol), 8 ml of H_2O_2(30%), 8 ml of acetic acid and 30 ml of methanol were heated under reflux for about six hours. Afterwards the methanol was removed under reduced pressure. The crude yellow coloured product was recrystallized from methanol.

### Refinement

The C-H H atoms were positioned with idealized geometry and refined isotropic with C-H = 0.93Å and *U*_iso_(H) = 1.2 times *U*_eq_(C); The H atoms of the amino groups was located in difference map and was refined isotropic with *U*_iso_(H)= 1.2 times *U*_eq_(N) and a N-H distance restrained to 0.90 Å.

### Crystal data

**Table:**

C_10_H_8_BrN_3_O	*F*(000) = 528.0
*M**_r_* = 266.09	*D*_x_ = 1.757 Mg m^−^^3^
Monoclinic, *P*2_1_/*n*	Mo *K*α radiation, λ = 0.71073 Å
Hall symbol: -P 2yn	Cell parameters from 2721 reflections
*a* = 13.402 (3) Å	θ = 2.4–26.8°
*b* = 5.3016 (10) Å	µ = 4.06 mm^−^^1^
*c* = 14.562 (3) Å	*T* = 298 K
β = 103.498 (3)°	Block, yellow
*V* = 1006.1 (4) Å^3^	0.52 × 0.13 × 0.11 mm
*Z* = 4	

### Data collection

**Table:**

Bruker SMART CCD area-detector diffractometer	1850 independent reflections
Radiation source: sealed tube	1263 reflections with *I* > 2σ(*I*)
graphite	*R*_int_ = 0.049
φ and ω scans	θ_max_ = 25.5°, θ_min_ = 1.9°
Absorption correction: multi-scan (*SADABS*; Bruker, 1997)	*h* = −16→15
*T*_min_ = 0.545, *T*_max_ = 0.650	*k* = −6→6
5867 measured reflections	*l* = −17→17

### Refinement

**Table:**

Refinement on *F*^2^	Primary atom site location: structure-invariant direct methods
Least-squares matrix: full	Secondary atom site location: difference Fourier map
*R*[*F*^2^ > 2σ(*F*^2^)] = 0.029	Hydrogen site location: inferred from neighbouring sites
*wR*(*F*^2^) = 0.054	H atoms treated by a mixture of independent and constrained refinement
*S* = 0.98	*w* = 1/[σ^2^(*F*_o_^2^) + (0.0029*P*)^2^ + 0.3286*P*] where *P* = (*F*_o_^2^ + 2*F*_c_^2^)/3
1850 reflections	(Δ/σ)_max_ = 0.001
139 parameters	Δρ_max_ = 0.40 e Å^−^^3^
1 restraint	Δρ_min_ = −0.28 e Å^−^^3^

### Special details

**Table:**

Geometry. All e.s.d.'s (except the e.s.d. in the dihedral angle between two l.s. planes) are estimated using the full covariance matrix. The cell e.s.d.'s are taken into account individually in the estimation of e.s.d.'s in distances, angles and torsion angles; correlations between e.s.d.'s in cell parameters are only used when they are defined by crystal symmetry. An approximate (isotropic) treatment of cell e.s.d.'s is used for estimating e.s.d.'s involving l.s. planes.
Refinement. Refinement of *F*^2^ against ALL reflections. The weighted *R*-factor *wR* and goodness of fit *S* are based on *F*^2^, conventional *R*-factors *R* are based on *F*, with *F* set to zero for negative *F*^2^. The threshold expression of *F*^2^ > σ(*F*^2^) is used only for calculating *R*-factors(gt) *etc*. and is not relevant to the choice of reflections for refinement. *R*-factors based on *F*^2^ are statistically about twice as large as those based on *F*, and *R*- factors based on ALL data will be even larger.

### Fractional atomic coordinates and isotropic or equivalent isotropic displacement parameters (Å^2^)

**Table:**

	*x*	*y*	*z*	*U*_iso_*/*U*_eq_	
Br1	0.92158 (2)	1.17815 (7)	−0.12095 (2)	0.06564 (15)	
C1	0.7817 (2)	1.1290 (5)	−0.11489 (19)	0.0432 (7)	
C2	0.7096 (2)	1.2903 (6)	−0.1629 (2)	0.0517 (8)	
H2	0.7262	1.4226	−0.1986	0.062*	
C3	0.6096 (2)	1.2463 (6)	−0.1558 (2)	0.0539 (8)	
H3	0.5567	1.3500	−0.1874	0.065*	
C4	0.5892 (2)	1.0499 (5)	−0.1021 (2)	0.0478 (7)	
H4	0.5224	1.0174	−0.0973	0.057*	
C5	0.6696 (2)	0.9003 (5)	−0.05497 (18)	0.0387 (7)	
C6	0.7173 (2)	0.5429 (5)	0.05848 (18)	0.0395 (7)	
C7	0.8226 (2)	0.5275 (5)	0.07141 (19)	0.0475 (7)	
H7	0.8566	0.6394	0.0401	0.057*	
C8	0.8775 (2)	0.3478 (6)	0.1303 (2)	0.0512 (8)	
H8	0.9484	0.3392	0.1390	0.061*	
C9	0.8273 (2)	0.1797 (6)	0.1766 (2)	0.0510 (7)	
H9	0.8636	0.0556	0.2158	0.061*	
C10	0.7240 (2)	0.1999 (6)	0.1637 (2)	0.0489 (7)	
H10	0.6898	0.0883	0.1950	0.059*	
H2A	0.5830 (5)	0.675 (5)	−0.0026 (18)	0.059*	
N1	0.76650 (17)	0.9372 (4)	−0.06143 (15)	0.0429 (6)	
N2	0.65001 (17)	0.7072 (4)	0.00308 (17)	0.0451 (6)	
N3	0.66939 (17)	0.3773 (4)	0.10671 (16)	0.0426 (6)	
O1	0.57060 (15)	0.3965 (4)	0.09841 (14)	0.0631 (6)	

### Atomic displacement parameters (Å^2^)

**Table:**

	*U*^11^	*U*^22^	*U*^33^	*U*^12^	*U*^13^	*U*^23^
Br1	0.0485 (2)	0.0779 (3)	0.0748 (2)	−0.01014 (19)	0.02297 (15)	0.0068 (2)
C1	0.0412 (17)	0.048 (2)	0.0399 (17)	−0.0028 (14)	0.0086 (13)	−0.0046 (15)
C2	0.052 (2)	0.050 (2)	0.0534 (19)	−0.0026 (16)	0.0119 (15)	0.0078 (16)
C3	0.053 (2)	0.053 (2)	0.0493 (19)	0.0087 (15)	0.0006 (15)	0.0044 (15)
C4	0.0362 (17)	0.0498 (19)	0.0561 (19)	0.0003 (15)	0.0085 (14)	0.0016 (16)
C5	0.0397 (17)	0.0356 (18)	0.0400 (16)	−0.0020 (13)	0.0077 (13)	−0.0041 (13)
C6	0.0430 (17)	0.0347 (17)	0.0404 (16)	−0.0026 (14)	0.0088 (13)	−0.0023 (14)
C7	0.0388 (17)	0.0476 (19)	0.0568 (19)	−0.0020 (14)	0.0128 (14)	0.0059 (16)
C8	0.0418 (17)	0.053 (2)	0.058 (2)	0.0003 (16)	0.0086 (14)	0.0043 (18)
C9	0.052 (2)	0.0442 (18)	0.0530 (19)	0.0030 (17)	0.0042 (14)	0.0027 (16)
C10	0.057 (2)	0.0437 (19)	0.0451 (18)	−0.0067 (17)	0.0110 (14)	0.0077 (16)
N1	0.0427 (15)	0.0405 (15)	0.0463 (15)	−0.0035 (11)	0.0121 (11)	0.0023 (12)
N2	0.0317 (13)	0.0451 (15)	0.0590 (15)	−0.0018 (12)	0.0112 (11)	0.0079 (13)
N3	0.0393 (15)	0.0444 (16)	0.0453 (14)	−0.0050 (12)	0.0125 (11)	−0.0004 (12)
O1	0.0411 (13)	0.0767 (17)	0.0725 (15)	−0.0049 (11)	0.0153 (11)	0.0112 (11)

### Geometric parameters (Å, °)

**Table:**

Br1—C1	1.916 (3)	C6—N3	1.374 (3)
C1—N1	1.325 (3)	C6—C7	1.382 (3)
C1—C2	1.356 (4)	C7—C8	1.374 (4)
C2—C3	1.388 (4)	C7—H7	0.9300
C2—H2	0.9300	C8—C9	1.383 (4)
C3—C4	1.367 (4)	C8—H8	0.9300
C3—H3	0.9300	C9—C10	1.357 (4)
C4—C5	1.384 (4)	C9—H9	0.9300
C4—H4	0.9300	C10—N3	1.351 (3)
C5—N1	1.338 (3)	C10—H10	0.9300
C5—N2	1.391 (3)	N2—H2A	0.899 (10)
C6—N2	1.372 (3)	N3—O1	1.305 (3)
			
N1—C1—C2	126.8 (3)	C8—C7—H7	119.8
N1—C1—Br1	114.7 (2)	C6—C7—H7	119.8
C2—C1—Br1	118.5 (2)	C7—C8—C9	120.1 (3)
C1—C2—C3	116.1 (3)	C7—C8—H8	120.0
C1—C2—H2	122.0	C9—C8—H8	120.0
C3—C2—H2	122.0	C10—C9—C8	118.5 (3)
C4—C3—C2	119.7 (3)	C10—C9—H9	120.7
C4—C3—H3	120.1	C8—C9—H9	120.7
C2—C3—H3	120.1	N3—C10—C9	121.9 (3)
C3—C4—C5	118.9 (3)	N3—C10—H10	119.0
C3—C4—H4	120.5	C9—C10—H10	119.0
C5—C4—H4	120.5	C1—N1—C5	116.0 (2)
N1—C5—C4	122.5 (3)	C6—N2—C5	129.2 (2)
N1—C5—N2	118.3 (2)	C6—N2—H2A	116.1 (17)
C4—C5—N2	119.1 (3)	C5—N2—H2A	114.2 (17)
N2—C6—N3	112.6 (2)	O1—N3—C10	120.2 (2)
N2—C6—C7	128.9 (3)	O1—N3—C6	119.2 (2)
N3—C6—C7	118.5 (3)	C10—N3—C6	120.5 (2)
C8—C7—C6	120.4 (3)		

### Hydrogen-bond geometry (Å, °)

**Table:**

*D*—H···*A*	*D*—H	H···*A*	*D*···*A*	*D*—H···*A*
N2—H2A···O1	0.90 (1)	2.12 (2)	2.542 (3)	108.(2)
